# Cashew reject meal in diets of laying chickens: nutritional and economic suitability

**DOI:** 10.1186/s40781-015-0051-7

**Published:** 2015-05-07

**Authors:** Taiwo O Akande, Akinyinka O Akinwumi, Taye O Abegunde

**Affiliations:** Department of Animal Science, Obafemi Awolowo University Ile-Ife, Ile-Ife, Osun State Nigeria; Department of Animal Nutrition and Biotechnology, Ladoke Akintola University of Technology, P.M.B. 4000 Ogbomoso, Oyo State Nigeria

**Keywords:** Cashew rejects meal, Chemical constituents, Economy, Laying hens, Performance

## Abstract

The present study investigated the nutritional and economic suitability of cashew reject meal (full fat and defatted) as replacement for groundnut cake (GNC) in the diets of laying chickens. A total of eighty four brown shavers at 25 weeks of age were randomly allotted into seven dietary treatments each containing 6 replicates of 2 birds each*.* The seven diets prepared included diet 1, a control with GNC at 220gkg^−1^ as main protein source in the diet. Diets 2, 3 and 4 consist of gradual replacement of GNC with defatted cashew reject meal (DCRM) at 50%, 75% and 100% on weight for weight basis respectively while diets 5, 6 and 7 consist of gradual inclusion of full fat cashew reject meal (FCRM) to replace 25%, 35% and 50% of GNC protein respectively. Each group was allotted a diet in a completely randomized design in a study that lasted eight weeks during which records of the chemical constituent of the test ingredients, performance characteristics, egg quality traits and economic indicators were measured. Results showed that the crude protein were 22.10 and 35.4% for FCRM and DCRM respectively. Gross energy of DCRM was 5035 kcal/kg compared to GNC, 4752 kcal/kg. Result of aflatoxin B_1_ revealed moderate level between 10 and 17 μg/Kg in DCRM and GNC samples respectively. Birds on control gained 10 g, while those on DCRM and FCRM gained about 35 g and 120 g respectively. Feed intake declined (P < 0.05) with increased level of FCRM. Hen day production was highest in birds fed DCRM, followed by control and lowest value (P < 0.05) was recorded for FCRM. No significant change (P > 0.05) was observed for egg weight and shell thickness. Fat deposition and cholesterol content increased (P > 0.05) with increasing level of FCRM. The cost of feed per kilogram decreased gradually with increased inclusion level of CRM. The prediction equation showed the relative worth of DCRM compared to GNC was 92.3% whereas the actual market price of GNC triples that of DCRM. It was recommended that GNC could be completely replaced by DCRM in layer’s diets in regions where this by product is abundant. However, FCRM should be cautiously used in diets of laying chickens.

## Background

The subject of feed resources and their utilization represents possibly the most compelling task facing producers and scientists in poultry industry. The attempt to mitigate high cost of feed has prompted the continuous search for alternatives or additional feedstuffs. This search for solutions is hinged on the concept of self-reliance which strives to achieve sustainability based on the use of indigenous resources. Cashew (*Anacardiaceae*) nut is receiving attention of scientist as a viable additional protein feed resource.

Cashew nut is an important industrial and export crop with wide distribution throughout the tropics in many parts of Africa and Asia [1]. Cashews rank third in world production of edible nuts that are traded globally and its nutritional values have long been recognized [2]. Global production was estimated by FAO at 4,439,960 metric tonnes and Nigeria, highest producer in Africa, produced about 950,000 metric tonnes representing 21.4% of the world total [3].

Cashew Nut Shell Liquid (CNSL), an important by-product of cashew industry with diverse use in friction linings, paints and varnishes, laminating and epoxy resins, foundry chemicals and as an intermediary of chemicals are gaining rapid commercial reputation. Recently, the extension of cashew nut from human consumption to the feeding of livestock has received attention of scientists [4-7] as a potential feedstuff. The upsurge in the consumption of the cashew nut has resulted in its large-scale production for local consumption and export due to the involvement of private entrepreneurs, Federal and State Governments, Cooperative societies and affluent farmers in cashew cultivation [8].

During processing, large quantities of the kennels are discarded because they are not suitable for sale as a result of bruises, damages, oiliness or because they are scorched during the drying process. It was estimated that up to about 30% of kernels may be lost in this manner depending on the quality of nuts. Although not suitable for sale, these reject cashew kernels has found application in animal feeding [3,5,6]. According to [9], cashew nut proteins are complete; having all the essential amino acids and a kilogramme of the nut yields about 6000 calories. It was earlier observed that young growing rats fed cashew reject meal had higher and protein efficiency ratio than those fed soybean meal while reject cashew kernel demonstrated weight gains superiority over groundnut cake and soybean meal when evaluated at a critical protein level with growing pigs [10,11].

The steady and high turnover from cashew production firm coupled with previous success reports are good indication that cashew kernel meal could be a viable feedstuff in animal feed [3,12-14]. However, there was paucity of information regarding the use of cashew nut in animal feed, the utilization of cashew nuts meal in diets of chickens has received little or no attention after over a decade of research.

This situation demands for concerted effort to develop cashew nut further as additional protein source for animals to minimize the incorporation of the highly priced conventional plant protein resources. Hence, the objectives of this study were to further examine nutritional value of cashew reject meal (full fat and defatted) and their economic implication in diets of laying chickens.

## Methods

### Sample collection and preparation

The experiment was carried out at the Poultry Unit, Teaching and Research Farm, Ladoke Akintola University of Technology, Ogbomoso, Oyo State, Nigeria. Cashew reject meal was purchased from Olam Nigeria Limited, Oyo State, Nigeria. The nuts after processing were graded according to quality. Those ones not suitable for human consumption are regarded as reject and were purchased for this study. Two batches were collected, one batch was defatted using hydraulic press to obtain defatted cashew reject meal (DCRM) and the second left undefatted as full fat cashew reject meal (FCRM).

### Birds management and experimental diets

A total number of 84 shaver pullets at 25 weeks of age were procured from a reputable farm and used for the study. Two tiers type battery cage housing was used to house the birds, 2 birds per cell. The birds were randomly allotted into seven dietary groups and each group subdivided into 6 replicates containing 2 birds each. Birds were stabilized for two weeks to adjust to the new environment before introduced to experimental diets which was supplied *ad- libitum.* All recommended vaccinations (ND and IBD) and preventive medication were administered accordingly. Normal daylight of 12 hr natural lighting regime was observed throughout the 10-week experimentation. Seven diets were prepared (Table 1) including a standard diet as control with groundnut cake as main source of protein. Diets 2, 3 and 4 consist of gradual replacement of GNC with DCRM at 50%, 75% and 100% weight for weight basis respectively while diets 5, 6 and 7 consist of gradual inclusion of FCRM to replace 25%, 35% and 50% of GNC protein respectively. Each diet was randomly allotted to a group of experimental birds.

**Table 1:** **Diet compositions for the experiment**

	**Control**	**Defatted cashew reject meal**			**Full fat cashew reject meal**		
**Composition,%**	**0%**	**50%**	**75%**	**100%**	**25%**	**37.5%**	**50%**
Maize	40.00	40.00	40.00	40.00	40.00	40.00	40.00
Groundnut cake	22.00	11.00	5.50	0.00	16.50	13.75	11.00
Corn bran	20.00	20.00	20.00	20.00	14.80	12.25	9.60
Palm kernel cake	5.00	5.00	5.00	5.00	5.00	5.00	5.00
Bone meal	3.00	3.00	3.00	3.00	3.00	3.00	3.00
Oystershell	7.00	7.00	7.00	7.00	7.00	7.00	7.00
Fish meal	2.00	2.00	2.00	2.00	2.00	2.00	2.00
Lysine	0.25	0.25	0.25	0.25	0.25	0.25	0.25
Methionine	0.25	0.25	0.25	0.25	0.25	0.25	0.25
Salt	0.25	0.25	0.25	0.25	0.25	0.25	0.25
^1^Premix	0.25	0.25	0.25	0.25	0.25	0.25	0.25
DCRM	-	11	16.5	22	-	-	-
FCRM	-	-	-	-	10.7	16.0	21.4
Total	100.00	100.00	100.00	100.00	100.00	100.00	100.00
***Calculated analysis, %***							
Crude Protein	17.50	16.75	16.40	16.00	17.05	16.75	16.45
Crude Fat	3.35	4.10	4.30	4.50	4.98	5.20	5.40
Crude Fibre	6.54	6.45	6.40	6.38	6.48	6.46	6.44
Metabolizable Energy, kcal/kg	2722	2768	2775	2782	2778	2806	2835

O- Oyster shell, DCRM- Defatted cashew reject meal, FCRM- Full fat cashew reject meal. ^1^Premix contain the following per kg diet: Vitamins A, 10,000 IU; D3,3,000 IU; E 8.0U; K, 2.0 mg; B6, 1.2 mg; B12, 0.12 mg; Niacin, 1.0 mg; Pantothenic acid, 7.0 mg; Folic acid, 0.6 mg; Choline, chloride, 500 mg; Minerals: Fe, 60 mg; Mn, 80 mg Mg, 100 mg; Cu, 8.0 mg; Zn, 50 mg; Co, 0.45 mg; I, 2.0 mg; Se, 0.1 mg.

### Data collection and analysis

The following data were collected: Chemical constituent of cashew reject meal: proximate composition, calorie level and some antinutritional factors; Performance and egg quality characteristics; some serum constituents and economic analysis. The relative worth was calculated from formular proposed by [15]. Cashew reject meal and groundnut cake as test samples were analyzed for proximate contents using [16] method. The total polyphenols as tannin equivalent was determine as described by [17]. The caloric value was determined using a bomb calorimeter. The phytin-phosphorus was determined and phytin content was calculated by multiplying the value of phytin-phosphorus by 3.55 [18]. The extracted aflatoxins were separated by thin-layer chromatography and the concentration of aflatoxin B_1_ was determined spectrophotometrically (R = 363 mm, V = 22, 000) by the method of [19]. All data collected were subjected to one way analysis of variance (ANOVA) using computer software package [20]. Significant means were separated by Duncan’s Multiple Range test of the same statistical software package.

## Results and discussion

### Chemical constituents of cashew reject meal

Table 2 shows the chemical composition of CRM. Its crude protein was 35.4%, affirming its status as high protein feedstuff. The value is higher than value obtained for coconut meal, palm kernel meal, linseed meal and Tung seed meal [18]. The value is comparable with value obtained for defatted castor seed meal, sunflower and rape seed meal [18,21]. The value is lower but closely related to those obtained for SBM and GNC. Crude fibre of deffatted CRM was 1.05% and similar to value reported by [22] which is substantially lower, when compared with other conventional protein sources such as GNC of which had 4.30%. The lower value obtained may be as a result of complete separation of the kernel during the shelling process since the roasted kernel was intended for human consumption.

**Table 2:** **Nutrient and antinutrient composition of cashew reject meal and groundnut cake**

**Composition, %**	**Defatted cashew reject meal**	**Full fat cashew R M**	**Groundnut cake**
Moisture	8.50	8.90	9.50
Crude protein	35.40	22.10	43.10
Crude fibre	1.05	0.90	4.30
Ether extract	15.10	40.23	6.00
Ash	5.45	3.73	5.51
Nitrogen free extract	34.50	24.04	31.80
Gross energy, kcal/kg	5035	6542	4752
Tannin %	1.51	1.02	5.80
Phytic acid %	0.52	0.50	0.75
Aflatoxin μg/Kg ppm	10.00	8.75	17.37

Gross energy derived from CRM was substantially higher, 5035 kcal/kg when compared with 4752 kcal/kg for GNC. The higher calory in CRM may be attributed to higher residual oil content because of mechanical defatting process that left the cake with about 15% residual oil.

The result of aflatoxin B_1_ reveals moderate level between 10 and 17 μg/Kg in the samples investigated. Considering the FDA tolerance level for total aflatoxin in food for human and poultry, the aflatoxin B_1_ levels in cashew nut meal is tolerable [23]. However, caution should be exercised against prolonged storage and feeding of fungi infected GNC in view of the health hazard associated with aflatoxin.

In poultry, tannin is known to interfere with digestion by displaying anti-trypsin and anti-amylase activity and complex with Vitamin B_12_ [24]. The lower tannin content, 1.5% observed for CRM compared to 5.8% in GNC suggest that nutrient availability in CRM based diets may be more secured than GNC. Although, phytic acid is a strong chelator forming protein and mineral-phytic acid complexes capable of reducing protein and mineral bioavailability in animals, the results here are moderate between 0.52 and 0.75% in the two samples, CRM and GNC respectively. These levels are tolerable and present no harm to laying hens as indicated in [25]. The analytical data indicate that the CRM could be an important alternative protein and energy contributors to compound animal feed in high producing region.

### Performance characteristics of laying birds fed cashew reject meal based diet

The results of performance traits were shown in Table 3. It has been established that animals eat in order to satisfy their energy requirement to promote growth, helps in cell formation and repairs [26]. Experimental birds increased their feed intake as the energy levels of the diet decreased. It was indicative that both defatted and full fat cashew reject meal contain more energetic component than their groundnut counterparts as shown by results of chemical analysis Table 2. The significantly (P < 0.05) lower feed intake recorded for the birds at 100% level of DCRM and 50% FCRM diets may suggest that the level of oil remaining in CRM is high enough to influence voluntary intake due high calories content. Meanwhile continuous and prolong consumption of high levels of fat may predispose the birds to prolapsed as a result of accumulation of fat in birds.

**Table 3:** **Performance, egg qualities and serum characteristics of layers fed cashew reject meal based diets**

	**Control**	**Defatted cashew reject meal**			**Full fat cashew reject meal**			
	**0%**	**50%**	**75%**	**100%**	**25%**	**37.5%**	**50%**	**SEM**
***Performance characteristics, g***								
Initial Live Wt	1565.00	1570.50	1480..00	1355.70	1443.80	1520.40	1505.50	65.55
Final Live Wt	1576.70^ab^	1586.70^a^	1496.00^c^	1400.00^c^	1500.00^bc^	1676.70^a^	1650.00^a^	80.10
Av. body wt gained	10.00^c^	15.10^c^	15.50^c^	44.00^b^	55.50^b^	155.20^a^	144 .00^a^	13.10
Hen day Prod. (%)	63.90^ab^	67.20^a^	68.90^a^	62.70^ab^	58.60^b^	55.80^b^	58.60^b^	5.00
Feed intake (g/day)	130^a^	140^a^	130^a^	110^c^	128^a^	125^ab^	115b^c^	7.30
Abdominal fat wt	4.34^c^	4.28^c^	5.60^b^	5.97^b^	5.62^b^	6.24^ab^	8.01^a^	0.55
Feed conversion R.	3.66	4.36	3.38	3.57	3.65	4.16	4.11	0.52
Cost of feed (₦)	59.71^a^	53.66^b^	50.64^bc^	47.51^c^	56.27^ab^	54.02^bc^	51.77^bc^	2.10
Cost/kg egg weight, ₦	218.53^ab^	244.69^a^	171.16^c^	169.61^c^	200.50^b^	204.93^b^	212.77^b^	11.50
***Egg quality characteristics***								
Egg weight, g	49.7	53.7	53.83	49.00	49.83	53.00	49.00	1.5
Shell thickness, mm	0.33	0.34	0.34	0.33	0.32	0.32	0.34	0.03
Yolk thickness, cm	1.56	1.58	1.59	1.62	1.56	1.61	1.65	0.25
Albumen thickness, cm	0.66	0.66	0.70	0.59	0.59	0.65	0.65	0.05
***Serum composition***								
Cholesterol, mg/dl	55.00	50.00	55.05	62.00	64.00	62.00	65.00	8.50
Protein, g/dl	4.20	3.65	3.95	3.70	3.60	3.70	4.15	0.35
Albumin, g/dl	2.40	2.20	2.00	2.30	2.10	1.90	2.30	0.25
Globulin, g/dl	1.80	1.45	1.95	1.40	1.50	1.80	1.85	0.55

Means with different superscripts within the same row differ significantly (P < 0.05).

Cost of GNC = ₦85.00, CRM = ₦25.00.

Cost/kg egg weight = feed cost Xfeed conversion ratio.

In terms of egg production, significant difference (P < 0.05) was observed across the treatments. Birds fed control diet had 64% production whereas those on DCRM had higher values (67%) and those on full fat diet had lower production 58%. The lower production percentage can be associated to the level fat in the diet consumed which did not translate into egg production instead it favor birds weight gain as noted in live weight Table 3. High values obtained for fat deposition in birds fed FCRM based diets can be linked to the higher fat content of the diets as these birds tends to have increased fat in the adipose tissue. Fat deposition is not desirable in laying hens because of the chances of prolapsed condition and possible decrease in egg production which may negatively influence the net returns to farmers. For egg weight, the values ranged from 49.00-53.33 g across the treatments with values slightly appreciating in CRM based diets.

Feed to gain ratio is a good indicator of biological efficiency of the diets [27]. The similarities (P > 0.05) among the diets indicate an equivalent biological efficiency. On the average however, birds on the CRM slightly appreciate in feed to gain ratio compared to those on control diet. This was also translated to the lower cost of feed/kg and feed cost/kg egg weight compared to control diet (Table 3).

### Serum components of laying birds fed cashew reject meal based diets

From Table 3, serum cholesterol shows no significant difference across dietary treatments (P > 0.05). This suggests that full fat soya bean meal; though high in fat do not contribute to cholesterol content or factors that influence its synthesis in laying hens. Cholesterol has been identified as an intermediate in metabolic pathways and important in homeostasis of healthy animals [28,29]. Serum protein is known for its functions in replacement of tissue proteins, buffer in acid–base balance and as transporter of constituents of blood such as vitamins, iron, copper, hormones, lipids and enzymes [30]. Any alteration in serum protein may impact on these functions and consequently affect the overall well being of the animals. Total protein, albumin and globulin content of the blood were the same across the treatment (P > 0.05) in this study. This means that there was no ill effect or rather there was no compromise in the health status of experimental laying hens. The result disagrees with findings of [31] who reported that serum protein of birds fed with the control diet and those fed with the cashew nut based diets were significantly affected.

### Economic analysis

The economic comparison take into account protein and energy which are usually the major nutrient cost in this diet, these nutrients give 85-90% estimation of overall economic worth. Also corn and soyabean meal are usually the major energy and protein sources in poultry diets, they can be used as basis for comparison [15].

In Table 4, the predicted cost compared to market price provides guidance to buyer on the relative worth of an ingredient. The relative worth of GNC and CRM compared to soyabean meal in this study are ₦97.25 and ₦90.00 respectively in terms of calories and crude protein estimates (calculated from prediction equation of [15]). In other words, GNC and DCRM are 97% and 90% worth respectively of soya bean meal. The actual market prices of these plant proteins SBM, GNC and CRM were ₦100.00, ₦85.00 and ₦25.00. It is very obvious that CRM had a comparable value worth with groundnut cake is substantially cheaper than the conventional groundnut cake.

**Table 4:** **Economic analysis of using CRM in place of GNC in diets of laying hens**

**Cost variables,** ₦	**Soyabean meal**	**Groundnut cake**	**Cashew reject meal**
Actual market price	100	85.00	25.00
Relative worth	100	97.25	90.00
Av. cost of feed/kg	-	59.71	50.61
Cost of feed/kg egg weight	-	218.53	200.50
Cost save per tonnage of feed	**-**		9,100
Cost save per tonnage of egg laid			18,030

Equation: Relative worth of CRM (with 35.4% CP and 2800 kcal) = 354x + 2800y.

Where × = 0.111 protein cost, y = 0.0183-energy cost.

Obtained from: maize (8.6% CP, 3300 kcal/kgME) and soyabean meal (48%CP, 2550 kcal/kgME) as reference energy and protein ingredients respectively. Given the cost prize for maize and soyabean at the time of the experiment -₦70.00 and ₦100.00 respectively.

Hence 86x + 3300y = 70------equation 1 and 480 × + 2550y = 100 -------equation 2.

The cost of feed per kilogram differs significantly (P < 0.05) across the treatments. The cost of feed was higher in control than diets containing both DCRM and FFCRM. The values of feed to gain ratio across the treatments and low feed cost of CRM diets reflects the biological and economical suitability of CRM when included in diet of laying birds. For every tonnage of feed compounded in this study, about ₦9,100.00 were saved when defatted cashew reject meal (CRM) was used to replace GNC. Moreso, for every tonnage of egg produced from laying hens, about ₦49,000.00 could be saved when CRM completely replaced GNC in the diet. The use of CRM in this study appears to justify the assertion by [32,33] that non-conventional feeding resources are capable of inducing savings by curtailing feed production costs. Apart from the fact that CRM was cheaper to GNC in terms of market value, its biological efficiency is commendable, with little anti nutritional compounds [15]. With more processing industries springing up and increased awareness in the export potentials of cashew nuts, more attention should shift to this economic tree.

## Conclusion

The use of both DCRM or FCRM has not in any way jeopardise the performance of laying birds in this study and the cost per unit of cashew nut compared to groundnut is significantly lower. Cashew reject meal has great potential of meeting the need for additional protein source, thus saving the nations millions of hard earned foreign exchange expended each year on the importation of groundnut from all part of the world. It was demonstrated that DCRM meal supported satisfactory performance of laying hens with low cost and can be incorporated in the diet of laying birds for complete replacement (i.e. 22%) of groundnut cake while FCRM should be cautiously used in laying birds as a result higher fat content.
